# Selective Inhibition by Ethanol of Mitochondrial Calcium Influx Mediated by Uncoupling Protein-2 in Relation to *N*-Methyl-D-Aspartate Cytotoxicity in Cultured Neurons

**DOI:** 10.1371/journal.pone.0069718

**Published:** 2013-07-16

**Authors:** Ryo Fukumori, Takeshi Takarada, Ryota Nakazato, Koichi Fujikawa, Miki Kou, Eiichi Hinoi, Yukio Yoneda

**Affiliations:** Laboratory of Molecular Pharmacology, Division of Pharmaceutical Sciences, Kanazawa University Graduate School, Kanazawa, Ishikawa, Japan; Chiba University Center for Forensic Mental Health, Japan

## Abstract

**Background:**

We have shown the involvement of mitochondrial uncoupling protein-2 (UCP2) in the cytotoxicity by N-methyl-D-aspartate receptor (NMDAR) through a mechanism relevant to the increased mitochondrial Ca^2+^ levels in HEK293 cells with acquired NMDAR channels. Here, we evaluated pharmacological profiles of ethanol on the NMDA-induced increase in mitochondrial Ca^2+^ levels in cultured murine neocortical neurons.

**Methodology/Principal Findings:**

In neurons exposed to glutamate or NMDA, a significant increase was seen in mitochondrial Ca^2+^ levels determined by Rhod-2 at concentrations of 0.1 to 100 µM. Further addition of 250 mM ethanol significantly inhibited the increase by glutamate and NMDA in Rhod-2 fluorescence, while similarly potent inhibition of the NMDA-induced increase was seen after exposure to ethanol at 50 to 250 mM in cultured neurons. Lentiviral overexpression of UCP2 significantly accelerated the increase by NMDA in Rhod-2 fluorescence in neurons, without affecting Fluo-3 fluorescence for intracellular Ca^2+^ levels. In neurons overexpressing UCP2, exposure to ethanol resulted in significantly more effective inhibition of the NMDA-induced increase in mitochondrial free Ca^2+^ levels than in those without UCP2 overexpression, despite a similarly efficient increase in intracellular Ca^2+^ levels irrespective of UCP2 overexpression. Overexpression of UCP2 significantly increased the number of dead cells in a manner prevented by ethanol in neurons exposed to glutamate. In HEK293 cells with NMDAR containing GluN2B subunit, more efficient inhibition was similarly induced by ethanol at 50 and 250 mM on the NMDA-induced increase in mitochondrial Ca^2+^ levels than in those with GluN2A subunit. Decreased protein levels of GluN2B, but not GluN2A, subunit were seen in immunoprecipitates with UCP2 from neurons with brief exposure to ethanol at concentrations over 50 mM.

**Conclusions/Significance:**

Ethanol could inhibit the interaction between UCP2 and NMDAR channels to prevent the mitochondrial Ca^2+^ incorporation and cell death after NMDAR activation in neurons.

## Introduction

Evidence that mitochondria are a key mediator of cell death through apoptotic and/or necrotic processes [1], [2] as well as an organelle essential for cellular respiration is accumulating in the literature. Cytosolic free Ca^2+^ ions would induce an opening of the mitochondrial permeability transition pore (mPTP) [3], [4] responsible for the increased permeability of mitochondrial outer membranes for different cytotoxic molecules, such as cytochrome C, apoptosis inducing factor, etc. [5], [6]. Accordingly, mPTP would mediate a disruption of the mitochondrial membrane potential (ΔΨ) for subsequent mitochondrial swelling to cell death. Calcium entry is shown to more easily occur into mitochondria after activation of *N*-methyl-D-aspartate (NMDA) receptor (NMDAR) channels rather than other Ca^2+^ gates, including kainate receptors and voltage-sensitive Ca^2+^ channels [7], [8].

The inhibition of Ca^2+^ transport into mitochondria protects neurons from cell death mediated by glutamate (Glu), suggesting that Glu-induced neuronal death requires Ca^2+^ entry into mitochondria [9]. In fact, mitochondrial dysfunction is a primary determinant of the fate of neurons exposed to Glu [10]. Reversible nuclear oxidative DNA damage occurs in cerebral cortical neurons in response to transient Glu receptor stimulation [11]. The mechanism seems to involve the mitochondrial calcium uniporter (mCU), which has been a conceptual carrier of cytoplasmic free Ca^2+^ ions into the matrix across inner membranes in mitochondria [12], [13]. Uncoupling proteins (UCPs) are proposed to be a candidate carrier for the influx of cytoplasmic Ca^2+^ ions toward the regulation of matrix Ca^2+^ levels in mitochondria [14], [15]. Recent studies have also identified molecular components as an mCU required for the influx of intracellular Ca^2+^ ions into mitochondrial matrix [16], [17].

We have shown that brief exposure to either Glu or NMDA leads to subsequent loss of cellular viability in cultured rat hippocampal neurons, without markedly affecting that in rat cortical neurons, along with more effective ΔΨ disruption in hippocampal neurons than in cortical neurons [18]. Reverse transcription polymerase chain reaction (RT-PCR) analysis revealed the absence of either *UCP1* or *UCP3* mRNA from cultured cortical and hippocampal neurons, while significantly higher expression levels are found in mRNA for both *UCP2* and *UCP4*, but not for *UCP5*, in vulnerable hippocampal neurons than in resistant cortical neurons [19]. Artificial overexpression of UCP2 leads to acceleration of the NMDA-induced increase in mitochondrial free Ca^2+^ levels determined by Rhod-2 and subsequent cytotoxicity, without affecting that in intracellular free Ca^2+^ levels determined by Fluo-3, in cultured human embryonic kidney (HEK)-293 cells with acquired functional NMDAR channels composed of GluN1/GluN2A and GluN1/GluN2B subunits [20].

On the other hand, the prevailing view is that NMDAR is at least in part responsible for the exhibition of different pharmacological profiles of the central depressant ethanol in the mammals [21], in addition to the involvement of both ionotropic and metabotropic receptors for gamma-aminobutyric acid [22]–[24]. For example, ethanol is shown to selectively inhibit the ion current induced by NMDA in a concentration-dependent manner at concentrations of 5 to 50 mM in cultured hippocampal neurons [25]. A significant inhibition by ethanol is seen in both Ca^2+^ influx and cGMP production after activation of NMDAR in cultured cerebellar granule neurons [26], while ethanol sensitivity is attributed to the status of the third and fourth transmembrane domains of both GluN1 and GluN2A subunits on functional NMDAR channels [27]. By contrast, a conditional knockout strategy clearly reveals the importance of GluN2B subunit rather than GluN2A subunit for the exhibition of acute and chronic pharmacological actions of ethanol in the murine brain [28], [29].

These previous studies prompted us to evaluate pharmacological properties of ethanol on the mitochondrial Ca^2+^ incorporation mediated by UCP2, as well as the cytotoxicity, seen after activation of NMDAR in cultured murine neocortical neurons and HEK293 cells with acquired functional NMDAR channels composed of different subunit compositions.

## Materials and Methods

### Materials

Both HEK293 and HEK293T cells were purchased from RIKEN Cell Bank (Saitama, Japan). The plasmid constructs *pcDNA1-GluN2A* and *pcDNA3.1-GluN1-1a* were generous gifts from Dr. Jon W. Johnson (Department of Neuroscience, University of Pittsburgh, Pittsburgh, PA, USA). The enhanced green fluorescent protein (EGFP) vector (*pEGFP-C2*) was purchased from Clontech (Mountain View, CA, USA). Poly-L-lysine, Hoechst33342, propidium iodide (PI), A23187 and NMDA were purchased from Sigma-Aldrich fine Chemicals (St. Louis, MO, USA). Antibodies against GluN1 and GluN2A subunits were supplied by Santa Cruz Biotechnology (Santa Cruz, CA, USA). Fluo-3 acetoxymethyl (AM) ester and rhodamine-2 (Rhod-2) AM ester were provided by Molecular Probes (Eugene, OR, USA). Dulbecco’s modified Eagle medium (DMEM) was obtained from Gibco BRL (Grand Island, NY, USA). Other chemical used were all of the highest purity commercially available.

### Preparation of Murine Cortical Neurons

This study was carried out in strict accordance with the recommendations in the Guide for the Care and Use of Laboratory Animals of the Japanese Society for Pharmacology. The protocol was approved by the Committee on the Ethics of Animal Experiments of Kanazawa University (Permit Number: AP-111873) with sustained efforts to minimize the number of animals used and their suffering. Primary neocortical neuronal cultures were obtained from 15-day-old embryonic ddY mice as described previously for rats [18], with minor modifications. In brief, cerebral neocortex was dissected from embryonic mouse brains, cleared of meninges and incubated with 0.25% trypsin in phosphate-buffered saline (PBS) at 37°C for 20 min. Tissue sediments were then mechanically triturated using a Pipetman with a 1000 µl-tip in the culture medium, followed by washing with culture medium and subsequent plating at a density of 7.5 × 10^5^ cells/cm^2^ on plastic dishes previously coated with 7.5 µg/ml poly-L-lysine in DMEM supplemented with 33 mM glucose, 2 mM glutamine, 100 U/ml penicillin, 100 µg/ml streptomycin, 5 mM HEPES, 13 mM sodium bicarbonate, 50 µg/ml apo-transferrin, 500 ng/ml insulin, 1 pM β-estradiol, 3 nM triiodothyronine, 20 nM progesterone, 8 ng/ml sodium selenite and 100 µM putrescine. Prior to each use, culture media were invariably filtered through a polyethersulfone membrane with a pore size of 0.2 µm. Cells were cultured in DMEM with the aforementioned supplementation for different days up to 8 days at 37°C in a 5% CO_2_/95% air humidified incubator with medium change every 3 days. Under these culture conditions, approximately 90% of cells were immunoreactive for the neuronal marker microtubules-associated protein-2 (MAP2) on immunocytochemical analysis using an antibody against MAP2.

### Procedures for Lentiviral Infection

The lentiviral backbone vector containing the ubiquitin C promoter used as an empty vector (EV) and three helper plasmids (*pRSV-REV*, *pMDLg/pRRE* and *vesicular somatitis virus G* (VSVG) protein-expressing plasmid) were kindly provided by Dr. Sudhof (Stanford University, Palo Alto, CA, USA). Full-length cDNA for mouse UCP2 was cloned into a lentiviral backbone vector produced as described previously [30]. Lentiviral vector, *pRSV-REV*, *pMDLg/pRRE* and *VSVG protein-expressing plasmid* were co-transfected into HEK293T cells at 10, 2.5, 5 and 3 mg of DNA per 56.7 cm^2^ of the culture area using the calcium phosphate method, respectively. Cells were cultured in DMEM supplemented with 2% fetal bovine serum (FBS) for 12 h after transfection, followed by replacement of culture medium with DMEM supplemented with 33 mM glucose, 2 mM glutamine, 100 U/ml penicillin, 100 µg/ml streptomycin, 5 mM HEPES, 13 mM sodium bicarbonate, 50 µg/ml apo-transferrin, 500 ng/ml insulin, 1 pM β-estradiol, 3 nM triiodothyronine, 20 nM progesterone, 8 ng/ml sodium selenite and 100 µM putrescine and subsequent further culture for an additional 48 h. Culture medium was then collected for centrifugation at 500 g for 5 min, followed by direct addition of the supernatant containing lentivirus particles to the culture medium for primary cultured neurons. Cultured neurons were usually infected with the lentiviral vector at 200 µl/500 µl for 24 h on Day 4 for the analysis on Day 8 unless indicated otherwise. All experimental steps were performed under the level II bio-safety conditions.

### Determination of Mitochondrial Free Ca^2+^ Levels

For determination of the mitochondrial free Ca^2+^ levels, we used the Ca^2+^-sensitive fluorescent dye Rhod-2 known to be accumulated into mitochondria due to its high cationic charge. In fact, Rhod-2 fluorescence is highly detected in areas completely merged with MitoTracker fluorescence [19]. In order to produce colorless and non-fluorescent dihydro Rhod-2 AM ester, which is a membrane-permeable form, Rhod-2 AM ester was incubated for 10 min with a particular amount of NaBH_4_ until the red color of Rhod-2 AM ester vanished according to the manufacturer’s instruction as described previously [31]. Dihydro Rhod-2 AM ester is permeable across cell membranes for the cleavage of AM ester and subsequent oxidization to the dye Rhod-2 for Ca^2+^-dependent fluorescence in the mitochondrial environment.

Cultured cells were washed once with recording medium containing 129 mM NaCl, 4 mM KCl, 1 mM MgCl_2_, 2 mM CaCl_2_, 4.2 mM glucose and 10 mM HEPES (pH 7.4), followed by incubation at room temperature for 60 min in recording medium supplemented with 0.02% Pluronic F-127 and 3 µM dihydro Rhod-2 AM. Culture medium was changed to recording medium, followed by exposure to either Glu or NMDA at different concentrations and subsequent determination of the fluorescence intensity every 1 min. Fluorescence intensity was normalized after the addition of the Ca^2+^ ionophore A23187 at 10 µM at the end of each experiment for quantitative analysis. Cells were invariably used within 1 to 5 h after these procedures for observation of the fluorescence visualized by a confocal laser-scanning microscope equipped with a helium-neon laser (LSM510, Carl Zeiss, Jena, Germany). Images were obtained using an objective lens with numeral apertures of 0.5 (Plan-Neofluar) for 20-fold magnification. Fluorescence images labeled with Rhod-2 were collected using an excitation wavelength of 543 nm. Parameters of illumination and detection were controlled digitally for consistent settings throughout the experiments. Two successive digital images were collected usually at 512×512 pixels in the same visual field. In order to confirm the accumulation in mitochondria, cells were exposed to Rhod-2 AM for 1 h, followed by staining with MitoTracker Green FM at 100 nM for 15 min at 37°C and subsequent washing 3 times for observation of cellular illumination by a fluorescence microscope to visualize mitochondria. Drugs were prepared in recording medium immediately before each use.

### Determination of Intracellular Free Ca^2+^ Levels

Cultured cells were washed with recording medium containing 129 mM NaCl, 4 mM KCl, 1 mM MgCl_2_, 2 mM CaCl_2_, 4.2 mM glucose and 10 mM HEPES (pH 7.4) once, followed by incubation at 37°C for 50 min in recording medium containing 30 nM Pluronic F-127 and 3 µM Fluo-3 AM [32]. Cultures were then washed with recording medium twice, followed by settlement for at least 1 h in the recording medium and subsequent placement in a confocal laser-scanning microscope for observation. Medium was changed once more, followed by exposure for 5 min to NMDA at different concentrations for determination of fluorescence image. The calcium ionophore A23187 was then added at 10 µM to obtain the maximal fluorescence for quantitative normalization. Fluorescence images obtained with Fluo-3 were collected using an excitation wavelength of 488 nm. The parameters of illumination and detection were digitally controlled to keep the same settings throughout the experiments. The data obtained were subjected to quantification by normalization on the basis of fluorescence intensity in cells exposed to A23187 at 10 µM. For quantitative analysis of A23187 fluorescence, images were quantified using ImageJ software (NIH, Bethesda, MD, USA) as the mean gray value in a visual filed selected at random 5 min after the addition of A23187 at different concentrations.

### Determination of Cellular Viability

Cellular viability was examined by double staining with the membrane permeable dye Hoechst33342 and the membrane impermeable dye PI for DNA as described previously [31]. Cultured neurons were exposed to Glu at concentrations from 10 to 100 µM for 1 h, followed by further culture for an additional 24 h and subsequent washing with PBS once. Cells were then incubated at room temperature for 10 min in PBS containing 10 µM Hoechst33342 and 10 µM PI. Cells were observed using an epifluorescent microscope (BZ-8100; Keyence, Osaka, Japan). The numbers of Hoechst33342-positive and PI-positive cells were individually counted in four different visual fields chosen at random per each well in a blinded fashion, for subsequent calculation of percentages of PI-positive cells over Hoechst33342-positive cells as an index of cell death.

### Orchestration of Acquired NMDAR Channels

In this study, we used rat NMDAR subunits cloned into expression vectors as described previously [33]. HEK293 cells were grown in DMEM supplemented with 5% FBS before transfection. In line with the optimization protocol [34], cells were transfected at 1∶3:4 ratios with *GluN1-1a* subunit, either *GluN2A* or *GluN2B* subunit and *UCP2-Flag* expression vectors by the calcium phosphate co-precipitation method in minimum essential medium with 5% FBS and 10 µM dizocilpine (MK-801), followed by further culture for an additional 24 h unless otherwise indicated. We also introduced the vector *pEGFP-C2* to monitor the transfection efficiency. Cells were rinsed with recording medium, followed by loading of either Fluo-3 AM for intracellular Ca^2+^ determination or Rhod-2 for mitochondrial Ca^2+^ determination. Cells were then exposed to NMDA at different concentrations as needed.

The transfection efficiency was over 50% as measured by the *pEGFP-C2* vector with cloned EGFP, while introduction of *GluN1/GluN2A* vectors led to a marked decrease in percentages of GFP-positive cells over Hoechst33342-positive cells from 52.9±1.1% to 9.0±1.3% during the culture for 48 h. However, the NMDAR blocker MK-801 significantly prevented the decreases induced by introduction of NMDAR subunits in survival ratios of cells transfected with *GluN1/GluN2A* (30.0±0.6%) vectors when added after transfection for subsequent culture. As Glu in culture medium could thus induce cell death during the culture for 48 h through activation of acquired NMDAR channels artificially orchestrated in HEK293 cells [33], we employed cells cultured in the presence of 10 µM MK-801 for 24 h after transfection in order to reduce the number of dead cells before exposure to NMDA as much as possible in this study. Similar protection procedures are employed with the competitive GluN2 subunit antagonist DL-2-amino-5-phosphonopentanoic acid in HEK293 cells after the transfection of both GluN1 and GluN2A subunits [34].

Under the experimental conditions employed here, marked expression was seen with corresponding subunit proteins in homogenates of cells transfected with each subunit expression vector on Western blotting analysis [32]. Both Glu and NMDA invariably increased the number of fluorescent cells due to Fluo-3 for the intracellular free Ca^2+^ levels in HEK293 cells transfected with both GluN1/GluN2A subunits, while neither Glu nor NMDA was effective in markedly increasing the fluorescence in cells transfected with either GluN1 or GluN2A subunit alone [33].

### Procedures for Immunocytochemistry

Cultured neurons were washed with PBS once, followed by fixation in 4% paraformaldehyde for 15 min. Cells were rinsed in PBS and then blocked in PBS containing 10% FBS and 0.1% Triton-X 100 for 1 h. Cells were incubated with antibodies overnight at 4°C (anti-Flag, 1∶500; anti-UCP2, 1∶200), followed by rinsing three times with PBS and subsequent reaction with appropriate fluorescent secondary antibodies for 1 h at room temperature.

### Procedures for Immunoprecipitation

Cultured cells were solubilized in the lysis buffer containing 1% Nonidet P-40, followed by incubation with the anti-UCP2 antibody for 16 h at 4°C. Immunoprecipitates were washed 3 times with 20 mM Tris-HCl buffer (pH 7.5) containing 137 mM NaCl, followed by boiling in the buffer containing 2% sodium dodecylsulfate (SDS). An aliquot was subjected to 2% SDS/20% polyacrylamide gel electrophoresis, followed by transfer to nitrocellulose membranes and subsequent analysis using an antibody against each GluN2 subunit as described previously [18].

### Data Analysis

Results are all expressed as the mean ± S.E. and the statistical significance was determined by the two-tailed and unpaired Students’ *t*-test or 2-way ANOVA test with the level of significance set at P<0.05.

## Results

### Mitochondrial Ca^2+^ Levels in Cultured Neurons

**Figure 1:**
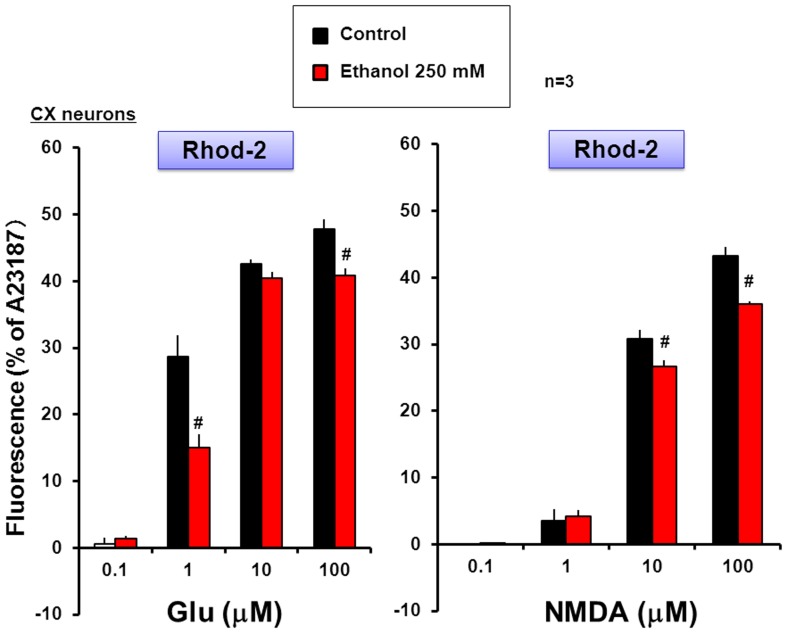
Effects of ethanol on Rhod-2 fluorescence in cultured neurons. Murine neocortical neurons were cultured for 8 days, followed by loading of Rhod-2 and subsequent cumulative exposure to Glu or NMDA at different concentrations in either the presence or absence of 250 mM ethanol. Values are percentages over the maximal fluorescence by A23187 in 3 independent determinations. ^#^P<0.05, significantly different from each control value obtained in cells not exposed to ethanol. Statistical significance was determined according to the Students’ *t*-testanalysis.

**Figure 2:**
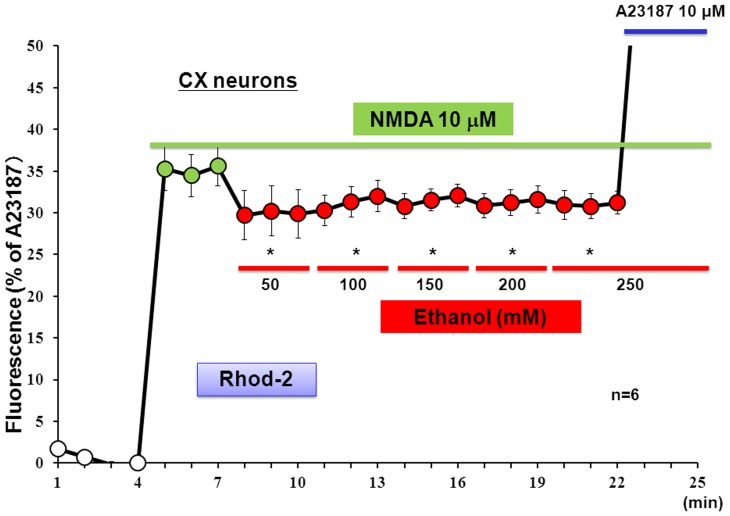
Effects of ethanol at different concentrations on Rhod-2 fluorescence in cultured neurons. Murine neocortical neurons were cultured for 8 days, followed by loading of Rhod-2 and subsequent cumulative addition of ethanol at different concentrations 3 min after the addition of 10 µM NMDA. Values are percentages over the maximal fluorescence by A23187 in 3 independent determinations. ^#^P<0.05, significantly different from the control value obtained in cells before the addition of ethanol. Statistical significance was determined according to the Students’ *t*-testanalysis.

Cortical neurons were cultured for 8 days, followed by loading of the mitochondrial Ca^2+^ dye Rhod-2 and subsequent exposure to Glu or NMDA at different concentrations for 5 min in either the presence or absence of 250 mM ethanol added 5 min before exposure to Glu and NMDA. Rhod-2 fluorescence was highly detected in intracellular locations completely merged with MitoTracker fluorescence as shown previously [19]. A concentration-dependent increase was seen in the Rhod-2 fluorescence in cortical neurons exposed to Glu (Figure 1, left panel) and NMDA (Figure 1, right panel) at concentrations from 0.1 to 100 µM, while further addition of 250 mM ethanol significantly inhibited the increases induced by Glu and NMDA. In neurons not exposed to Glu or NMDA, ethanol did not markedly affect the basal fluorescence level below detection. For evaluation of the concentration dependency, cultured neurons were exposed to a fixed concentration of NMDA, followed by cumulative addition of ethanol at final concentrations of 50 to 250 mM for determination of Rhod-2 fluorescence. In cortical neurons exposed to 10 µM NMDA, ethanol was invariably effective in significantly inhibiting the increased Rhod-2 fluorescence at concentrations over 50 mM up to 250 mM after cumulative addition in a manner independent of the concentrations used (Figure 2).

### Lentiviral Overexpression of UCP2 in Cultured Neurons

**Figure 3:**
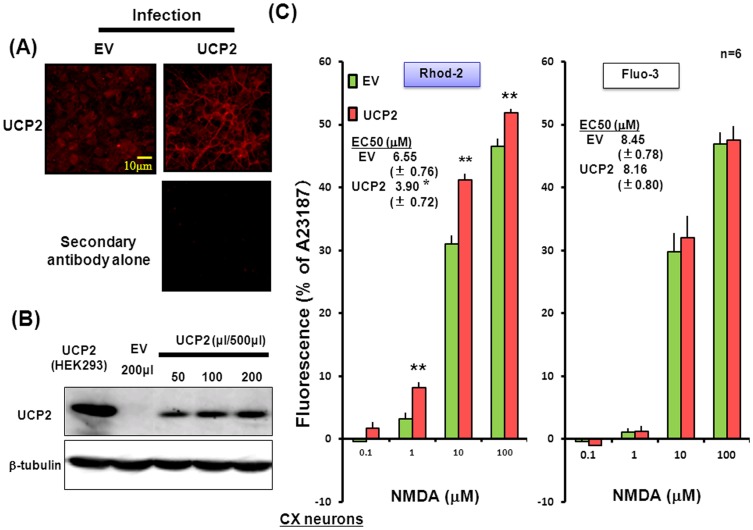
Lentiviral overexpression of UCP2 in cultured neurons. Murine neocortical neurons were cultured for 4 days, followed by infection with lentiviral UCP2 or EV expression vector at 50 to 200 µl/500 µl and subsequent further culture for an additional 4 days. Cells were then subjected to determination of UCP2 protein expression on (A) immunocytochemistry and (B) immunoblotting analyses. Cells were also loaded with either (C) Rhod-2 or (D) Fluo-3, followed by cumulative addition of NMDA at concentrations of 0.1 to 100 µM. Values are percentages over the maximal fluorescence by A23187 in 6 independent determinations. **P<0.01, significantly different from each control value obtained in cells with *EV* alone. Statistical significance was determined according to the Students’ *t*-testanalysis.

To confirm the involvement of UCP2 in the mitochondrial free Ca^2+^ increase mediated by activation of acquired NMDAR channels in HEK293 cells [20], murine fetal neocortical neurons were infected with a lentiviral expression vector of *UCP2* for stable overexpression. Immunocytochemical (Figure 3A) and Western blotting (Figure 3B) analyses clearly revealed the stable high expression of UCP2 protein in cultured neocortical neurons infected with the lentiviral *UCP2* vector at concentrations over 50 µl/500 µl. In cultured neurons infected with the lentiviral vector at 200 µl/500 µl, NMDA was significantly more effective in increasing Rhod-2 fluorescence for determination of mitochondrial free Ca^2+^ levels (Figure 3C, left panel), but not Fluo-3 fluorescence for determination of the intracellular free Ca^2+^ levels (Figure 3C, right panel), in a concentration-dependent manner than in neurons transfected with EV. In fact, overexpression of UCP2 led to a significant decrease in EC50 values (µM) for NMDA on Rhod-2 fluorescence from 6.55±0.76 to 3.90±0.72 without significantly affecting those on Fluo-3 fluorescence (8.45±0.76 vs. 8.16±0.80).

### Pharmacological Profiling of Ethanol in Cultured Neurons with UCP2 Overexpression

**Figure 4:**
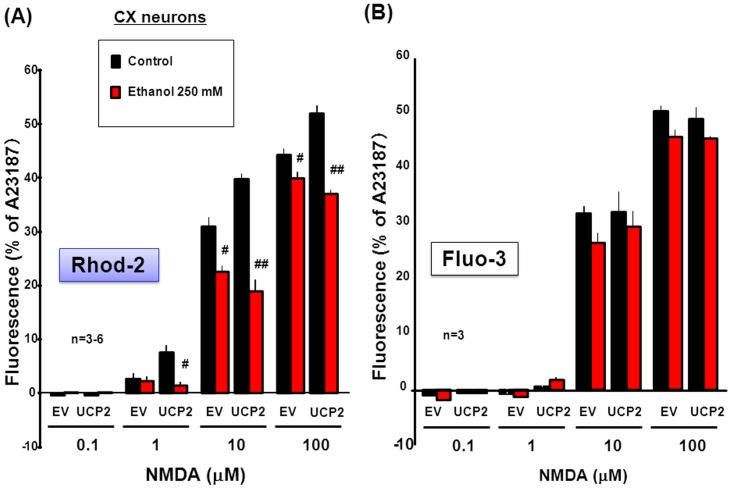
Effects of ethanol on Rhod-2 and Fluo-3 fluorescence in cultured neurons with UCP2 overexpression. Murine neocortical neurons were cultured for 4 days, followed by infection with lentiviral UCP2 or EV expression vector and subsequent further culture for an additional 4 days. Cells were then loaded with either (A) Rhod-2 or (B) Fluo-3, followed by cumulative addition of NMDA at concentrations of 0.1 to 100 µM in either the presence or absence of 250 mM ethanol. Values are percentages over the maximal fluorescence by A23187 in 3–6 independent determinations. ^#^P<0.05, ^##^P<0.01, significantly different from the value obtained in cells not exposed to ethanol. Statistical significance was determined according to the 2-way ANOVA test analysis.

In neurons transfected with EV, 250 mM ethanol significantly inhibited the concentration-dependent increase in Rhod-2 fluorescence by NMDA at concentrations of 0.1 to 100 µM (Figure 4A). In neurons with overexpressed UCP2, NMDA was significantly more effective in increasing Rhod-2 fluorescence at concentrations of 1 to 100 µM in a manner highly sensitive to the inhibition by 250 mM ethanol than in neurons with EV. However, ethanol failed to significantly affect the concentration-dependent increase by NMDA in Fluo-3 fluorescence in a fashion irrespective of overexpression of UCP2 (Figure 4B).

**Figure 5:**
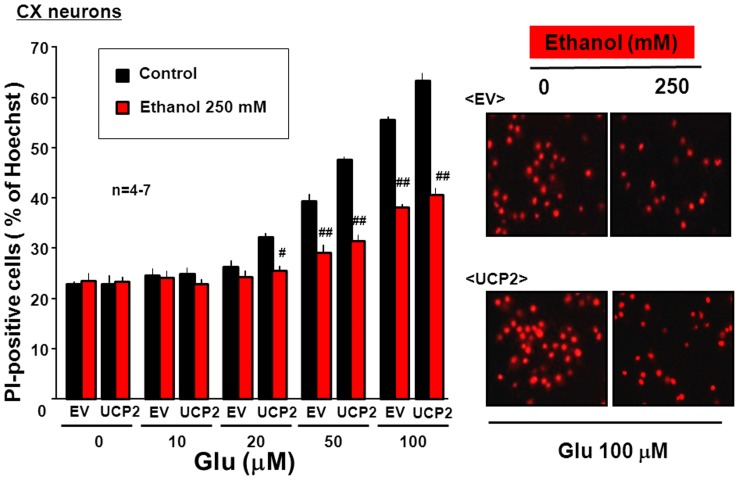
Effects of ethanol on cellular survival in cultured neurons with UCP2 overexpression. Murine neocortical neurons were cultured for 4 days, followed by infection with lentiviral UCP2 or EV expression vector and subsequent further culture for an additional 4 days. Cells were then exposed to Glu at concentrations of 10 to 100 µM for 1 h in either the presence or absence of 250 mM ethanol, followed by further culture for an additional 24 h and subsequent DNA staining with Hoechst33342 and PI for determination of cellular viability. Values are percentages of PI-positive cells over Hoechst33343-positive cells in 4–7 independent determinations. ^#^P<0.05, ^##^P<0.01, significantly different from the value obtained in cells not exposed to ethanol. Statistical significance was determined according to the 2-way ANOVA test analysis.

To confirm the positive correlation between mitochondrial free Ca^2+^ levels and cytotoxicity in HEK293 cells with acquired NMDAR channels as shown previously [20], cultured neocortical neurons were infected with a lentiviral vector of either *EV* or *UCP2*, followed by brief exposure to Glu at concentrations of up to 100 µM for 1 h and subsequent further culture for an additional 24 h for double staining with Hoechst33342 and PI to calculate the percentage of dead cells over total cells. In cells with EV, brief exposure to Glu induced a significant increase in the number of dead cells stained with PI in a concentration-dependent manner at concentrations of 50 to 100 µM (Figure 5). Overexpression of UCP2 was significantly more effective in increasing the number of dead cells stained with PI in cultured neurons briefly exposed to Glu at concentrations of 20 to 100 µM, whereas ethanol significantly alleviated percentages of dead cells to individual similar levels after brief exposure to Glu in neurons transfected with *EV* or *UCP2*. In particular, ethanol significantly diminished the increased number of PI-positive cells after brief exposure to 20 µM Glu in neurons with UCP2 overexpression without significantly altering that in neurons transfected with *EV*.

### Overexpression of UCP2 in HEK293 Cells with Acquired NMDAR Channels

**Figure 6:**
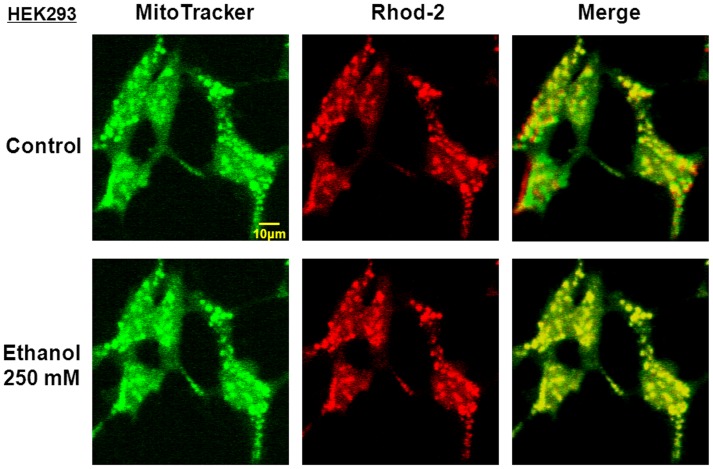
Effect of ethanol on morphological features of HEK293 cells. Cultured HEK293 cells were loaded with Rhod-2 along with MitoTracker green, followed by washing with recording medium and subsequent exposure to 250 mM ethanol for 20 min for observation under confocal laser scanning microscope. Typical pictures are shown in this figure with similar results in 3 independent experiments.

**Figure 7:**
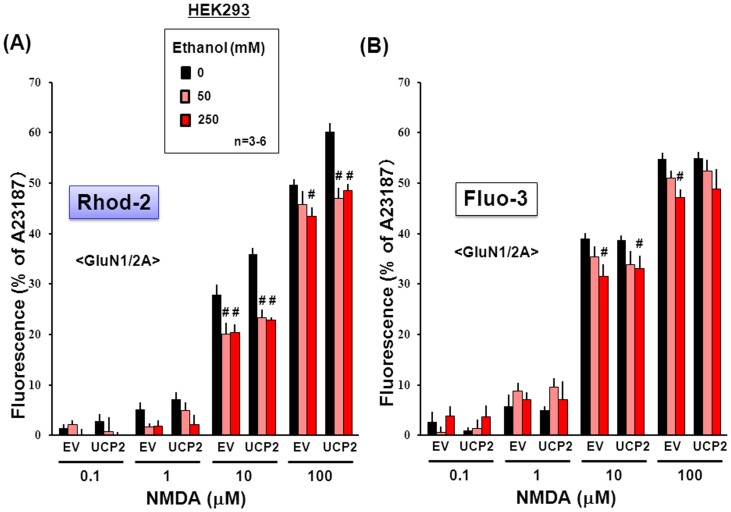
Effects of ethanol on Rhod-2 and Fluo-3 fluorescence in HEK293 cells with GluN1/GluN2A subunits. HEK293 cells were transfected with expression vectors of GluN1 and GluN2A subunits along with Flag-UCP2, followed by further culture for an additional 24 h and subsequent loading of either (A) Rhod-2 or (B) Fluo-3. Cells were cumulatively exposed to NMDA at 0.1 to 100 µM in either the presence or absence of ethanol at different concentrations. Values are percentages over the maximal fluorescence by A23187 in 3–6 independent determinations. ^#^P<0.05, significantly different from the value obtained in cells not exposed to ethanol. Statistical significance was determined according to the 2-way ANOVA test analysis.

In HEK293 cells transfected with a *Flag-UCP2* vector, but not with a *Flag-EV* vector, high co-localization was found with immunoreactivities for both anti-Flag and anti-UCP2 antibodies in intracellular locations positive to the staining with MitoTracker Green [20]. *In vitro* addition of 250 mM ethanol did not markedly alter morphological features of both MitoTracker and Rhod-2 fluorescence in HEK293 cells (Figure 6). Cells were thus transfected with expression vectors of *GluN1* and *GluN2A* subunits together with or without the *Flag-UCP2* vector, followed by loading of either Fluo-3 or Rhod-2 and subsequent exposure to NMDA at a concentration range of 0.1 to 100 µM in either the presence or absence of 50 and 250 mM ethanol. In cells with overexpression of both UCP2 and NMDAR, NMDA was significantly more effective in increasing the fluorescence intensity of Rhod-2 accumulated into mitochondria in a concentration-dependent manner at concentrations of 10 to 100 µM than in those with EV and NMDAR overexpression (Figure 7A). Ethanol at 50 and 250 mM significantly inhibited the NMDA-induced increase in Rhod-2 fluorescence in HEK293 cells with UCP2 overexpression to the level similar to that in cells with *EV*. In contrast to the fluorescence of Rhod-2, NMDA significantly increased the fluorescence intensity of Fluo-3 in a concentration-dependent manner at concentrations of 1 to 100 µM in cells expressing GluN1/GluN2A subunits to a similar extent irrespective of stable UCP2 overexpression (Figure 7B). Ethanol at 250 mM significantly inhibited the increase in Fluo-3 fluorescence by 100 µM NMDA in cells with *EV* only, but induced similarly significant inhibition of the increased Fluo-3 fluorescence in cells exposed to 10 µM NMDA irrespective of UCP2 overexpression.

**Figure 8:**
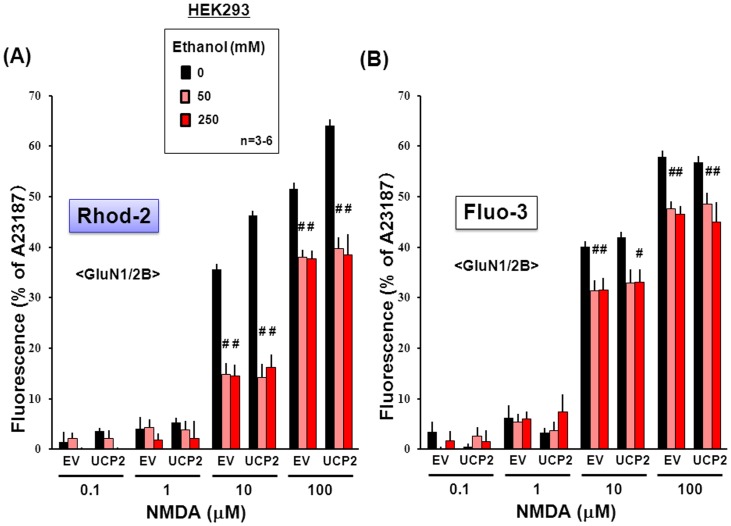
Effects of ethanol on Rhod-2 and Fluo-3 fluorescence in HEK293 cells with GluN1/GluN2B subunits. HEK293 cells were transfected with expression vectors of GluN1 and GluN2B subunits along with Flag-UCP2, followed by further culture for an additional 24 h and subsequent loading of either (A) Rhod-2 or (B) Fluo-3. Cells were cumulatively exposed to NMDA at 0.1 to 100 µM in either the presence or absence of ethanol at different concentrations. Values are percentages over the maximal fluorescence by A23187 in 3–6 independent determinations. ^#^P<0.05, significantly different from the value obtained in cells not exposed to ethanol. Statistical significance was determined according to 2-way ANOVA test analysis.

**Figure 9:**
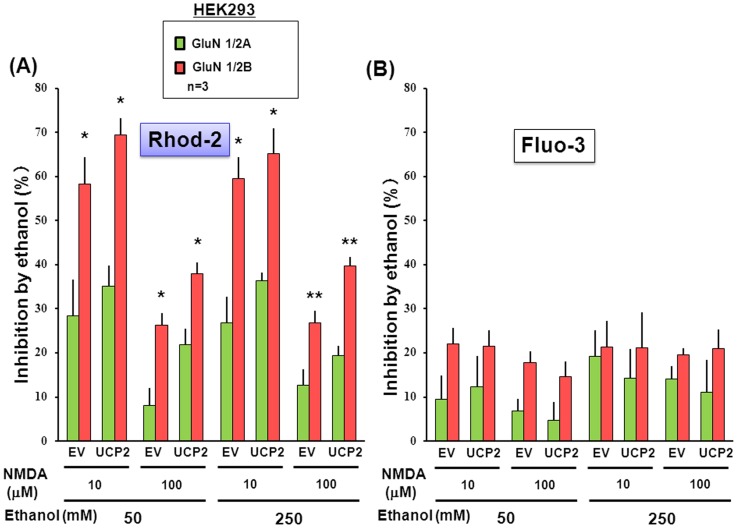
Comparison between inhibitions by ethanol on acquired NMDAR channels with GluN2A and GluN2B subunits. HEK293 cells were transfected with different NMDAR subunits along with Flag-UCP2, followed by further culture for an additional 24 h and subsequent loading of either (A) Rhod-2 or (B) Fluo-3. Cells were cumulatively exposed to NMDA at 0.1 to 100 µM in either the presence or absence of 50 and 250 mM ethanol. Values are percentages of the inhibition by ethanol in 3–6 independent determinations. *P<0.05, **P<0.01, significantly different from each control value obtained in cells with GluN2A subunit. Statistical significance was determined according to the 2-way ANOVA test analysis.

In order to evaluate the selectivity for a particular subunit composition, similar experiments were done in HEK293 cells transfected with *GluN1* and *GluN2B* subunits in either the presence or absence of the *UCP2* vector. Although overexpression of UCP2 led to a significantly more effective increase by NMDA at 10 to 100 µM in the fluorescence of Rhod-2 (Figure 8A) without affecting that of Fluo-3 (Figure 8B), ethanol drastically inhibited the increased Rhod-2 fluorescence at concentrations of 50 and 250 mM in cells with UCP2 overexpression to the level found in cells without UCP2 overexpression. In addition, ethanol was similarly effective in significantly inhibiting the NMDA-induced increase in Fluo-3 fluorescence at 50 and 250 mM irrespective of overexpression of UCP2. The inhibition by ethanol was significantly more efficient at 50 and 250 mM in cells with GluN2B subunit than in those with GluN2A subunit throughout the NMDA concentration range used from 10 to 100 µM in a manner irrespective of UCP2 overexpression for Rhod-2 fluorescence (Figure 9A), while ethanol failed to differentially inhibit the NMDA-induced increase in Fluo-3 fluorescence in cells overexpressing either GluN2A or GluN2B subunit at the individual concentrations used (Figure 9B).

**Figure 10:**
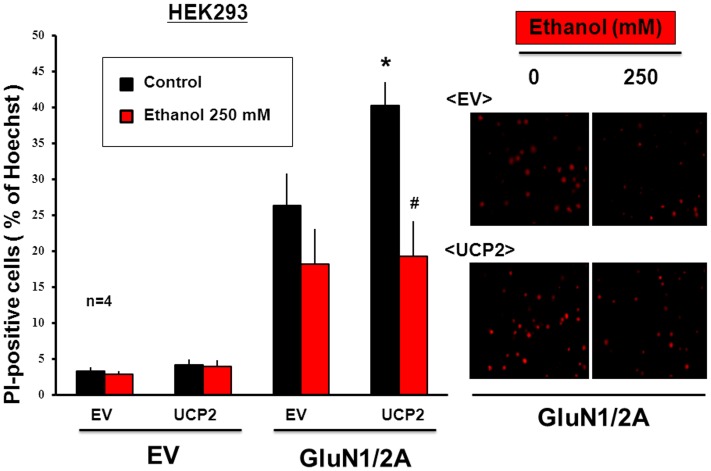
Effects of ethanol on cellular survival in HEK293 cells with GluN1/GluN2A subunits. HEK293 cells were transfected with expression vectors of GluN1 and GluN2A subunits along with Flag-UCP2, followed by further culture for an additional 24 h in either the presence or absence of 250 mM ethanol and subsequent DNA staining with Hoechst33343 and PI for determination of cellular viability. Values are percentages of PI-positive cells over Hoechst33343-positive cells in 4 independent determinations. *P<0.05, significantly different from each control value obtained in cells with *EV* alone. ^#^P<0.05, significantly different from the value obtained in cells not exposed to ethanol. Statistical significance was determined according to the Students’ *t*-testanalysis.

Cells were next cultured for an additional 24 h after the transfection with *NMDAR* subunit and *UCP2* expression vectors, and stained with the membrane permeable dye Hoechst33342 and the membrane impermeable dye PI for DNA to determine cellular viability. Artificial expression of GluN1/GluN2A subunits led to an increase in the number of dead cells stained with PI in cultured HEK293 cells in particular visual fields selected at random under microscope, while further overexpression of UCP2 significantly increased the number of PI-positive cells in cultured HEK293 cells with GluN1/GluN2A subunits, but not in those with *EV* in place of NMDAR subunits (Figure 10). In the presence of acquired NMDAR channels composed of GluN1/GluN2A subunits, moreover, ethanol was exclusively effective in significantly inhibiting the increase in the number of PI-positive cells upon UCP2 overexpression.

### Interaction between GluN2 Subunits and UCP2

**Figure 11:**
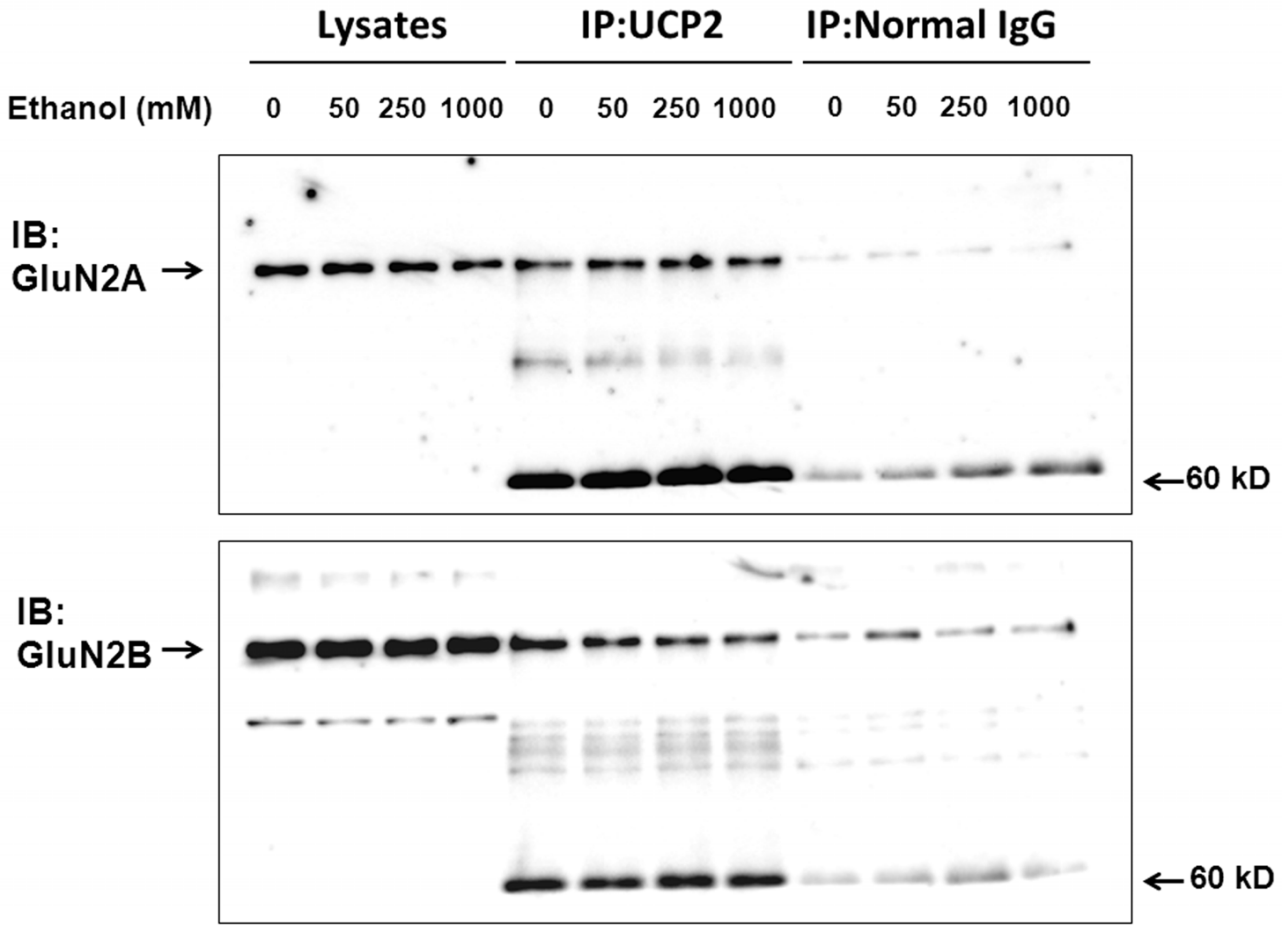
Effects of ethanol on interaction between GluN2 subunits and UCP2. Murine neocortical neurons were cultured for 8 days, followed by exposure to ethanol at concentrations of 50 mM to 1 M for 20 min before immunoprecipitation (IP) with the anti-UCP2 antibody for detection of immunoreactive GluN2 subunits on immunoblotting (IB). Typical pictures are shown with similar results in 2 independent experiments.

In HEK293 cells with overexpression of both Flag-UCP2 and NMDAR, immunoreactive GluN1 subunit was invariably detected in immunoprecipitates with the anti-Flag antibody from lysates of cells with overexpression of both UCP2 and NMDAR, but not from those with overexpression of NMDAR alone [20]. To analyze the underlying mechanism for the selective inhibition by ethanol for a particular GluN2 subunit, neocortical neurons cultured for 8 days were exposed to ethanol at concentrations of 50 mM to 1 M for 20 min, followed by solubilization by lysis buffer and subsequent immunoprecipitation with the anti-UCP2 antibody for immunoblotting detection of GluN2A and GluN2B subunits. Brief exposure to ethanol was found to decrease the immunoreactive GluN2B subunit levels at concentrations over 50 mM, without markedly affecting the GluN2A levels, in immunoprecipitates with UCP2 from cell lysates (Figure 11).

## Discussion

The essential importance of the present findings is that ethanol was more effective in significantly inhibiting the increased fluorescence of Rhod-2, but not that of Fluo-3, after activation of NMDAR in cultured murine neocortical neurons with overexpressed UCP2 than in those without UCP2 overexpression. Similar differential effectiveness was seen for the protection by ethanol against the increased neurotoxicity after brief exposure to Glu in cultured neurons with regard to UCP2 overexpression. To our knowledge, this is the first direct demonstration of the selective inhibition by ethanol of the promotion of both Rhod-2 fluorescence and neurotoxicity in cultured murine neocortical neurons exposed to NMDA *in vitro*. The current findings that lentiviral UCP2 overexpression significantly increased the mitochondrial free Ca^2+^ levels determined by Rhod-2 imaging without significantly affecting the intracellular free Ca^2+^ levels determined by Fluo-3 imaging in cultured neurons give strong support to the idea that UCP2 is really responsible for the incorporation of cytosolic free Ca^2+^ into mitochondrial matrix, in addition to mCU recently identified as a carrier for mitochondrial Ca^2+^ influx [16], [17]. In HEK293 cells with acquired functional NMDAR channels, moreover, UCP2 overexpression promotes the influx of Ca^2+^ into mitochondrial matrix rather than cytosolic spaces with concomitant exacerbation of the cytotoxicity after brief exposure to NMDA, without affecting the mitochondrial Ca^2+^ influx and the cytotoxicity after exposure to the calcium ionophore A23187 [20]. The possible involvement of mCU in mechanisms underlying the mitochondrial Ca^2+^ influx and neurotoxicity after NMDAR activation in neurons, however, remains to be elucidated in future studies. Thus, the idea that neuroprotection by ethanol is solely reflective of anything specific to UCP2 activity is not fully convincing.

In UCP2-null mice with permanent middle cerebral artery occlusion, significant ameliorations are seen in the infarct size, the number of apoptotic cells and lipid peroxidation levels in the peri-infarct areas [35]. In liver mitochondria from UCP2-null mice, Ca^2+^ sequestration occurs in a manner insensitive to a mitochondrial Ca^2+^ transport inhibitor [14]. Overexpression as well as silencing analysis clearly reveals the absolute requirement of both UCP2 and UCP3 isoforms for the incorporation of cytoplasmic free Ca^2+^ into mitochondria in response to physiological stimuli [14], [15]. The contribution of UCP2/3 is demonstrated to the transport of Ca^2+^ into mitochondria as mCU [36]. By contrast, increased UCP2 expression is shown to prevent cell death induced by seizure, hydrogen peroxide and nitric oxide in PC12 cells [37] and damage by tumor necrosis factor-α in hypothalamic neurons [38]. In transgenic mice with constitutive overexpression of UCP2 in the hippocampus, a robust reduction is seen in the loss of CA1 pyramidal neurons after seizures, suggesting neuroprotective properties of UCP2 in a mouse model of epileptic seizures [37]. Taking into consideration the physiological role of UCP2 as an uncoupler in mitochondrial energy synthesis, it is conceivable that cellular energy demands would at least in part determine whether UCP2 is neurotoxic or neuroprotective in a particular situation.

**Figure 12:**
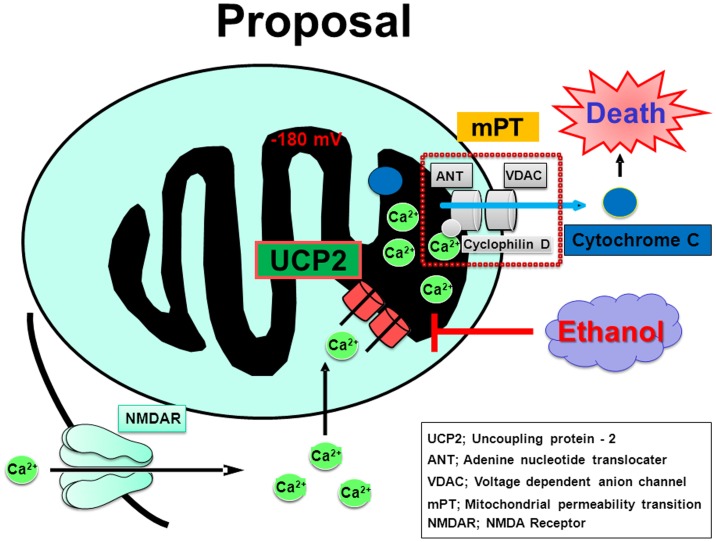
Proposed model of inhibition by ethanol of mitochondrial Ca^2+^ influx. Activation of NMDAR would lead to the incorporation of cytosolic Ca^2+^ into mitochondrial matrix in a manner relevant to UCP2 expressed at inner membranes toward the orchestration of mPTP responsible for the leakage of a variety of cytotoxic molecules in neurons. Ethanol could inhibit the mitochondrial Ca^2+^ influx occurred after the interaction between UCP2 and NMDAR channels through an unidentified mechanism.

The present data that ethanol significantly inhibited the increased Fluo-3 fluorescence seen after exposure to NMDA in HEK293 cells without UCP2 overexpression are in agreement with previous findings that ethanol inhibits both Ca^2+^ influx and cGMP production induced by NMDAR activation in cultured cerebellar granule neurons (Hoffman et al., 1989). Since the Ca^2+^ dye Fluo-3 would label intracellular free Ca^2+^ even in mitochondria, the possible detection of both cytosolic and mitochondrial Ca^2+^ levels in previous studies using this type of Ca^2+^-sensitive dye is not ruled out so far. In the present study, by contrast, the use of Rhod-2 has an advantage of the preferential detection of mitochondrial free Ca^2+^ levels due to its high cationic property required for the selective accumulation into this hyperpolarized organelle. Accordingly, our findings argue in favor of an idea that UCP2 plays a pivotal role in the transportation of cytosolic Ca^2+^ into mitochondria toward consequential orchestration of mPTP responsible for the leakage of a variety of cytotoxic molecules in neurons as shown in Figure 12. The predominance of functional NMDAR channel expression would be thus a determinant of the cellular vulnerability mediated by mitochondria to overflowed extracellular Glu amongst neurons, astroglia, microglia and oligodendroglia in the brain. Taken together, mitochondrial Ca^2+^ could play a dual role in the mechanisms relevant to the exacerbation or alleviation of cellular dysfunctions in a manner dependent on expression profiles between NMDAR and UCP2, in addition to the sorts of insults and cell types.

We have for the first time demonstrated that immunoreactive GluN1 subunit is invariably detected in immunoprecipitates with the antibody against Flag in a manner irrespective of the type of GluN2 subunits expressed in HEK293 cells with acquired NMDAR channels and overexpressed Flag-UCP2 [20]. One possible but hitherto unproven interpretation is that surface GluN1 subunit could directly or indirectly interact with mitochondrial UCP2 in HEK293 cells under artificial expression conditions. This interpretation would account for the predominant neurotoxicity seen after activation of NMDAR channels rather than different ligand-gated channels permeable to Ca^2+^. The fact that UCP2 is originally found as a proton transporter responsible for uncoupling of respiration from oxidative phosphorylation in mitochondria [39], [40] gives rise to an idea that cellular dysfunctions may involve a mechanism associated with the deteriorated energy synthesis after ΔΨ disruption mediated by the interaction between GluN1 subunit and UCP2 in cells with functional NMDAR channels permeable to Ca^2+^. Furthermore, the cytoplasmic C-terminal tail of NMDAR interacts with a variety of proteins within the postsynaptic density (PSD), notably the membrane-associated guanylate kinase family including PSD-95 [41], whereas PSD-95 plays an important role in NMDAR-mediated synaptic plasticity through a mechanism related to modulation of the localization, clustering and subunit expression profile [42]–[44]. Although the possibility that the interaction may be merely derived from the artificial expression of UCP2 and NMDAR subunits in non-neuronal cells is not ruled out, a recent study has demonstrated the expression of a Ca^2+^ transport protein with features similar to NMDAR in mitochondria of the rat brain [45].

It should be emphasized that ethanol was significantly more effective at a concentration range of 50 to 250 mM in inhibiting the NMDA-induced mitochondrial Ca^2+^ incorporation in acquired NMDAR channels composed of GluN2B subunit rather than in those of GluN2A subunit, along with similarly less potent inhibition of an intracellular Ca^2+^ increase after activation of NMDAR. In fact, GluN2B subunit is shown to be more crucial for the pharmacological featuring of ethanol than GluN2A subunit in the murine brain [28], [29]. The present findings that decreased GluN2B levels, but not GluN2A levels, were seen in immunoprecipitates with UCP2 from lysates of cultured murine neocortical neurons exposed to ethanol are favorable for the proposal that ethanol would inhibit the mitochondrial Ca^2+^ influx mediated by NMDAR in a manner preferentially associated with negative modulation of the interaction between UCP2 and NMDAR channels containing GluN2B subunit. Considering the preference to mitochondrial Ca^2+^ influx, coupled with the protection of cellular viability and the selectivity to GluN2B subunit seen after a brief exposure to ethanol for 3 to 60 min, it is unlikely that ethanol elicits the aforementioned actions through a strong cytotoxic property at an extraordinarily high concentration irrelevant to usual pharmacological activities in comparison with blood levels. Almost similar *in vitro* pharmacological profiles between ethanol at 50 and 250 mM in this study are not contradictory to this idea. Elucidation of the significance of the preference to the mitochondrial Ca^2+^ influx after the interaction between UCP2 and GluN2B subunit in different central actions of ethanol, however, should await further experimentation in future studies.

### Conclusion

It thus appears that ethanol may protect neurons from the cytotoxicity mediated by UCP2 after activation of NMDAR through a mechanism relevant to the adjustment of mitochondrial free Ca^2+^ levels. Elucidation of the possible molecular mechanisms underlying selective inhibition of the interaction between NMDAR and UCP2 would give a clue for novel strategies to ameliorate neuronal abnormalities related to malfunctions of NMDAR channels in patients with mild and/or severe alcoholism.
